# The case for policy-relevant conservation science

**DOI:** 10.1111/cobi.12444

**Published:** 2014-12-24

**Authors:** David C Rose

**Affiliations:** Department of Geography, University of CambridgeDowning Place CB2 3EN, Cambridge, United Kingdom email dcr31@hermes.cam.ac.uk

**Keywords:** boundary work, evidence-based conservation, evidence-informed policy, framing, science–policy interface, conservación con base en evidencias, interconexión ciencia-política, marco, política informada con evidencias, trabajo fronterizo

## Abstract

Drawing on the “evidence-based” (Sutherland et al. 2013) versus “evidence-informed” debate (Adams & Sandbrook 2013), which has become prominent in conservation science, I argue that science can be influential if it holds a dual reference (Lentsch & Weingart 2011) that contributes to the needs of policy makers whilst maintaining technical rigor. In line with such a strategy, conservation scientists are increasingly recognizing the usefulness of constructing narratives through which to enhance the influence of their evidence (Leslie et al. 2013; Lawton & Rudd 2014). Yet telling stories alone is rarely enough to influence policy; instead, these narratives must be policy relevant. To ensure that evidence is persuasive alongside other factors in a complex policy-making process, conservation scientists could follow 2 steps: reframe within salient political contexts and engage more productively in boundary work, which is defined as the ways in which scientists “construct, negotiate, and defend the boundary between science and policy” (Owens et al. 2006:640). These will both improve the chances of evidence-informed conservation policy.

El Caso para la Ciencia de la Conservación con Relevancia Política

**Resumen:**

A partir del debate “con base en evidencia” (Sutherland *et al*. 2013) versus “informado con evidencia” (Adams & Sandbrook 2013), debate que se ha vuelto prominente en la ciencia de la conservación, argumento que la ciencia puede ser influyente si mantiene una referencia dual (Lentsch & Weingart 2011) que contribuya a las necesidades de quienes hacen la política a la vez que mantiene un rigor técnico. En línea con dicha estrategia, los científicos de la conservación cada vez reconocen más la utilidad de construir narrativas con las cuales pueden mejorar la influencia de sus evidencias (Leslie et al. 2013; Lawton & Rudd 2014). Sin embargo, sólo contar historias rara vez es suficiente para influir sobre la política; en su lugar, estas narrativas deben ser políticamente relevantes. Para asegurar que la evidencia sea persuasiva junto con otros factores en un proceso complejo de fabricación de políticas, los científicos de la conservación pueden seguir dos pasos: rediseñar el marco de trabajo a partir de contextos políticos salientes y participar con mayor productividad en el trabajo fronterizo, que se define como los métodos con los cuales los científicos “construyen, negocian y defienden la frontera entre la ciencia y la política” (Owens et al. 2006:640). Estos pasos incrementarán la oportunidad de políticas de conservación informadas con evidencias.

## Introduction

An evidence-based approach to conservation, prominent in the field of conservation science, has been criticized for assuming a direct, instrumental transfer of knowledge to policy (Adams & Sandbrook 2013). Additionally, criticism has also been directed at scientists who focus on the shortcomings of policy makers to explain lack of research impact. For example, Sutherland et al. (2013) argue that conservation evidence will be more influential if policy makers understand “20 points” about science. However, others argue that the very concept of an evidence-based approach suffers from a lack of nuanced insights from social science and specifically insights from policy analysis (Agrawal & Ostrom 2006). Foremost among these critical authors, Tyler (2013) has chided Sutherland et al. (2013) for focusing on the inadequacy of policy makers while failing to address the way in which science is produced and communicated; rather Tyler encourages scientists to see the benefits of understanding processes of policy making. In recognizing that evidence is, therefore, just one factor in a complex decision-making process, Adams and Sandbrook (2013) suggest that conservation scientists should adopt an evidence-informed mindset which involves an understanding of how policy is formed. This is a better term to describe science–policy interactions because it helps divert attention away from always producing more and better science in line with a technical-rational mindset of decision making and instead directs attention toward a scenario in which end products can be made more efficient.

Embroiled within such debates about how to increase the impact of research, there have been several attempts to document the value of telling stories in conservation biology, as part of an approach based on the idea that evidence alone is rarely influential. For example, Leslie et al. (2013) argue that conservation and storytelling can go “hand-in-hand” and usefully call for a greater acknowledgement of the synergies between science and storytelling. Yet their research fails to draw sufficiently on interpretive policy analysis, a body of research exploring how policy makers and other actors in policy debates interpret evidence and construct meaning (Hajer 1995), and this contributes to the provision of little guidance on the art of policy-relevant storytelling. Similarly, Lawton and Rudd (2014) developed a “narrative policy framework,” but could offer additional practical advice about what scientists could do to improve impact.

Whilst acknowledging the potential value of storytelling, I sought to delve further into modes of policy formation. I aim to make a contribution to the ways in which conservation scientists seek to influence policy. Consequently, I have attempted to offer lessons for scientists across a wide spectrum of roles, particularly university researchers and scientists within conservation NGOs who wish to ensure that their evidence makes a difference in driving policy. Doing so adds to calls for greater understanding of, and engagement with, policy processes on the part of conservation scientists through the absorption of 2 ideas: reframe evidence, where possible, within salient political contexts and engage more productively in boundary work, which is defined as the ways in which scientists “construct, negotiate, and defend the boundary between science and policy” (Jasanoff 1990; Owens et al. 2006:640).

## Constructing a Compelling Conservation Narrative

One example of how conservation scientists might begin to tell effective stories is provided by van Bommel and van der Zouwen (2013), who argue that a good scientific narrative consists of 3 parts: a beginning which introduces the idea and sets the scene; a middle which fleshes out a problem; and an end which provides a solution which is uplifting or inspiring or which highlights the disturbing consequences of taking no action.

The storyteller must also find ways to hook the reader into the narrative and tie together empirical data and theoretical concepts throughout (van Bommel & van der Zouwen 2013). These authors also note that a balance must be struck between adequately addressing 2 questions: so what (i.e., why does the evidence matter?) and, latterly, did it really happen (i.e., is the evidence accurate or is the story representative of wider issues?)?

Storytelling is a useful tool through which to make conservation science more accessible to policy makers because it presents scientific arguments in an understandable way to nonexperts. Telling stories is an essential part of influencing the policy-making process, and the influence of stories can be enhanced by narrating good news (Balmford 2012). However, employing this strategy with limited attention paid to the needs of decision makers is inadequate. Thus, the framework developed by van Bommel and van der Zouwen should be treated with some caution because it fails to engage adequately with theories of policy formation.

**Figure 1:**
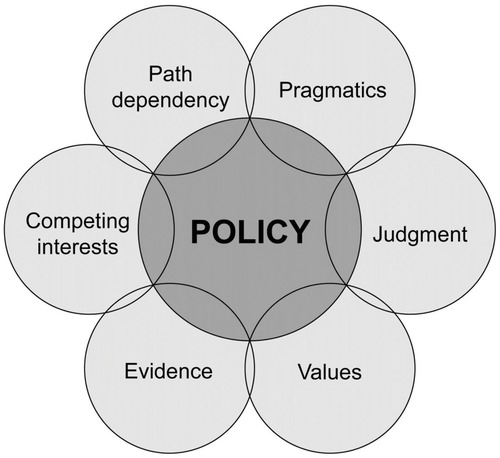
Factors that interact with the policy-making process, including competing interests, values, and path dependency (an attachment to historical ways of working which could constrain the use of new ideas) (adapted from Davies 2004).

Theorists of the policy process have shown that decisions are seldom made on the basis of evidence alone, instead they highlight the complexity of decision making (Owens 2012). This complexity is created because policy makers are faced with competing demands, indeed forming decisions based on a range of factors illustrated in Fig.1. These factors include values, judgment, pragmatics, path dependency, and other considerations. This complicated process of decision-making can also involve the selective political use of evidence (Owens 2012). Here, it is important to make a distinction between overt political manipulation of evidence and the legitimate processes of governing in a democracy, in which it is to be expected that scientific evidence will be among the many considerations that typically need to be taken into account. In line with democratic decision making, therefore, the use of science by policy makers in all areas of regulatory policy is mediated through interaction with other demands. Therefore, it is useful for conservation scientists to consider that evidence can usually only ever inform policy alongside other factors (Adams & Sandbrook 2013) which might be more politically salient at any one time.

If one accepts that the policy process is affected by the variety of variables highlighted in Fig.1 (and more besides), then it is clear that the ability of evidence alone to solve environmental problems is limited (Thompson & Warburton 1985; Sarewitz 2004; Rose 2014*a*); thus, scientists would do well to present their findings, and tell stories, in a way that allows evidence to be persuasive. This will not be achieved by making the evidence better or by creating narratives without consideration of political context. Instead, scientists should consider the competing goals of policy makers and incorporate knowledge of salient contexts as the overriding driver of van Bommel and van der Zouwen's (2013) 3-point plan.

## Framing and Policy-Relevant Advice

Framing draws boundaries around an issue, and the process determines what can be included in and excluded from a debate (Rein & Schön 1991; Palmer 2012). How an issue is framed, therefore, becomes crucial to deciding how policy makers interpret ideas; Riker (1986) argues that many of the most significant shifts in political life are caused by reframing the issues at stake. The work of Kingdon (2003) on government agenda setting illustrates that periods of favorable policy conditions (or “policy windows”) can be created if several process streams converge to support an idea. He argues that radical policy change can occur where policies, problems, and politics join together. In other words, if knowledge fits within pre-conceived pet ideas whilst solving a salient problem and within a context of receptive politics, evidence has the potential to be influential.

The power of reframing an issue is exemplified by a campaign in the European Union to ban the trade in wild birds, led by the Royal Society for the Protection of Birds (RSPB). The RSPB's campaign initially lacked policy relevance because they framed the narrative in terms of animal welfare, and this failed to influence decision makers. Ultimately, it was found that “the arguments about animal welfare and nature conservation were almost irrelevant” (Avery 2012:238).

The RSPB reframed their argument

[w]hen an imported South American parrot died, apparently from bird flu, we [RSPB] recognized that there was a chance to get the bird trade banned for reasons completely different from those that had motivated the RSPB for so long (Avery 2012:238).

The RSPB then argued their case for a ban on the basis of protecting human health because trading in wild birds could spread avian flu farther, a story that suddenly had a hook with a social, political, and even economic dimension. This re-telling quickly led to an EU ban. This example shows that conservation scientists can reframe the meaning of their evidence so that it speaks more directly to a salient political context.

Identifying hooks outside of nature conservation is vital to increase the saliency of scientific advice, and chances to do this could be missed by focusing on producing more evidence. Seizing opportunities to argue differently can be productive, further illustrated by Balmford's (2012) assessment of water management in South Africa. In this case, conservation biologists discovered the substantial threat invasive plant species (e.g., Monterey pine [*Pinus radiata*]) posed to native biodiversity and found that the water consumption patterns of these non-native species threatened draining water catchments. After struggling to influence policy initially, conservation scientists experienced a substantial research impact by persuading the government to set into motion a widespread non-native removal plan. Balmford (2012) argues that scientific evidence became influential because it was used to approach the problem of non-native plant species from a range of angles. In presenting knowledge to the government, it was clearly argued that human well-being and an ability to tackle social inequality were inextricably linked to ecosystem health. In 2012, Working for Water employed around 25,000 people in 300 projects to eradicate non-native species across South Africa, providing jobs for some of the poorest people in the country whilst achieving conservation benefits.

In line with these examples, a former conservation director of the RSPB argues that “it's the economic and social aspects [of policy making] that are most important—not the environmental ones” (Avery 2012:243). This is clearly illustrated, for example, by polls conducted for the 2014 European Union elections in which climate change and the environment fell far behind issues relating to the economy, immigration, and crime, in terms of electoral importance (EU Commission 2013). Protecting nature, therefore, is rarely likely to be the priority of most policy makers, and scientists would, therefore, benefit from presenting evidence in such a way as ensures conservation contributes to other issues.

## Achieving Policy Relevance for Conservation

It is true that there is relatively little well-developed theory of policy advice for scientists to follow (Owens 2011). However, drawing inspiration from the astute reframing of the RSPB wild bird campaign and Working for Water discussed previously, it is possible to recognize the value of discursively narrating policy-relevant knowledge wherever possible. I argue that 2 ideas could be embraced that increase the political salience of conservation evidence and more importantly that these points can be easily and efficiently employed by scientists.

## Reframing within salient political contexts

It is useful to offer lessons about how conservation scientists can harness the skills required for politically astute reframing of evidence. I attempt specifically to make my advice accessible to all conservation scientists, whether directly involved in policy discussions or not.

Being a conservation scientist does not mean one is not capable of keeping up with changes in political context (Young et al. 2014), for most governments hold pet ideas, many of which are widely known. In the case of the Conservative party, the major part of the U.K. Coalition Government which came to power in 2010, it was widely reported that localist forms of governance were supported; thus, conservationists might have seized upon this as a framework with which to argue for nature. In the vision of Takacs (1996), scientists could begin to take chances to protect nature by constructing stories not akin to traditional arguments. To this end, evidence must have a “dual reference” (Lentsch & Weingart 2011, 7), being scientifically sound and politically acceptable where possible and converging with pressing ideas (Kingdon 2003). This is perhaps an area in which conservation scientists miss opportunities (although the idea of ecosystem services, for example, has been seized upon); they sometimes rely on the strength of evidence alone and the persuasiveness of protecting nature for nature's sake, a value not shared by everyone.

One potential avenue to consider is the continued attachment of conservation objectives to protecting valuable ecosystem services or solving other mainstream political issues, such as climate change (these salient issues change over time) (Jørgensen et al. 2014; Young et al. 2014). Doing so may, of course, require more work to identify and measure ecosystem services vital for social well-being. However, in addressing concerns about adopting a policy-relevant approach, there are risks. For example, placing a price on nature in the form of ecosystem services necessitates a social construction of value (Vira & Adams 2009), and this might sometimes be less than the economic benefits of destroying nature. Furthermore, the RSPB achieved short-term success in terms of affecting a ban on trade of wild birds and highlighted that animal welfare is less salient than other framings, potentially undermining ethical arguments to protect nature. However, harnessing an awareness of political context does not necessarily mean we must “sell out on nature” (McCauley 2006). Instead, policy-relevant evidence can be deployed alongside arguments to protect nature for its own sake, adding to the toolbox available to conservation scientists who wish to influence policy.

Following on from the premise of reframing evidence, it is further important to help conservation biologists gain useful skills to identify pressing political issues more often, and it is vital to identify techniques that are relatively easy to learn. This latter point is important because Sutherland et al. (2013:335) argue that “it is unrealistic to expect substantially increased political involvement from scientists,” not necessarily discouraging the search for greater policy relevance, but questioning the practicality of doing so.

However, an analysis of advice offered by Young et al. (2014) illuminates several practical and efficient means of identifying salient policy contexts. These authors suggest a number of different approaches, such as organizing workshops attended by scientists and policy makers to allow joint framing, advocating better links with government science advisers, and promoting more interdisciplinary research. Moving on from these widely documented ideas, Young et al. (2014) also promote job shadowing and wider cross-reviewing of papers submitted to academic journals, both of which have much to offer. A short period of following a policy maker and witnessing the daily demands of her or his work would be an enlightening experience for many scientists, particularly those in training, and could facilitate a greater awareness of the reception of scientific evidence once it is deployed in policy debates. In regards to the South African eradication of non-native species, one can see the value of such skills. Here, the ecologists “didn't restrict themselves to thinking like ecologists; they thought like humans” (Balmford 2012:85). In so doing, the conservation scientists had the wit to “see problems through others’ eyes” (Balmford 2012:67) and placed the emphasis away from concerns over favored plant species and toward issues of substantial importance for policy makers. Gaining a first-hand appreciation of relevant issues by understanding the mechanics of the work of policy maker is necessary to see conservation issues through their eyes. Cross-reviewing might also lead to increased sharing of knowledge between, for example, policy analysts and conservation scientists.

It is encouraging to witness successful practices already underway in universities that are training the next generation of conservationists. Conservation postgraduate students in one group at the University of British Columbia, for example, are required to produce policy briefs for nearly every piece of research produced, whether it is a research paper, a popular science article, or blog. This involves training in how to write effective briefs in 500 words or so and practice in honing this technique over time to consolidate the importance of always seeing how their work fits into wider policy debates (an important and necessary philosophy even if a piece of work is not directly meant for policy makers).

### Engaging More Productively in Boundary Work

Drawing upon Gieryn's (1983) seminal research on boundary work, Cash et al. (2002:1) define boundaries as “socially constructed and negotiated borders between science and policy [and] between disciplines.” Gieryn (1983) referred to boundaries in a defensive sense, describing how scientists engaged in boundary work to keep out intellectual activities deemed to be pseudoscientific. However, later scholars of science and technology studies acknowledged that boundaries can be fluid (as did Gieryn), but also found that they are open to a constructive interpretation (Jasanoff 1990; Bijker et al. 2009; Rose 2014*b* ). In the context of increasing the impact of conservation research, Young et al. (2014) consider the value of boundary work yet do not recognize the full potential of a constructive interpretation. Instead, they focus on how scientists might link boundaries to bridge the communication gap between science and policy. This may be achieved by presenting evidence using “boundary objects” (Star & Griesmer 1989). These objects can take numerous forms such as documents, concepts, narratives, or models which are understandable and acceptable to both scientists and policy makers. For example, Turnhout et al. (2008) argue that ecological indicators can be employed by conservation scientists as a boundary object to fashion increased co-operation and better communication between actors in both social realms.

However, it is vital for scientists to understand that they (like policy makers) have agency to define a boundary's location so that its very position can be strategically changed. This constructive interpretation of boundary work moves beyond defending the discipline of science from interference by nonscientific sources to further identifying that better communication alone is sometimes inadequate to facilitate a positive research impact. Although not widely discussed in the conservation literature, Swart and van Andel (2008) describe how ecologists engaged in constructive boundary work in controversies surrounding cockle fishing in the Dutch Wadden Sea. Swart and van Andel found that (2008:86) a number of scientists departed from “the classic rule of focusing on avoiding type 1 errors” and instead stressed the importance of societal considerations. Therefore, the very process of producing policy-relevant science can be a form of constructive boundary work, which seeks to extend the reach of scientific knowledge in order for science to be influential in the political realm.

Of course, engaging in boundary work might initially be uncomfortable for scientists (Jasanoff 2013), who consider that the credibility of the scientific enterprise is undermined by any link with the policy-making process (Breining 2012). [Correction added after online publication on January 9, 2014: “comfortable” replaced with “uncomfortable” in the previous sentence.] Pullin and Knight (2012:4), for example, strongly condemn an “entrepreneurial free-for-all effort to promote individual studies to the policy community,” and other conservation scientists prefer to keep faith with a defensive approach to boundary work, rather than seeking to extend the reach of science (Morecroft et al. 2014; Rose 2014*b*). Yet, in conservation science, engagement with the policy process is desirable because conservation attempts to achieve an objective that is extrinsic to science itself, notably the protection of nature in practice. Furthermore, Cook et al. (2013) have argued convincingly that ‘boundary science’ does not necessarily see scientific legitimacy and credibility sacrificed for saliency.

## Producing Policy-Relevant Evidence

Conservation biology might, therefore, usefully measure the suitability of a research product not by scientific quality alone, but also by its applicability to the needs of policy makers (de Wit 2011). Conservation scientists could productively produce evidence which has a dual reference (Lentsch & Weingart 2011), presenting it “in a form that is not only plausible but persuasive” (Sandercock 2003:19). In the first instance, presenting information in the form of an understandable story is useful to engage nonexperts, but policy relevance is vital to hold their attention and make an impact. Thus, in recognizing that scientific knowledge is interpreted by policy makers, conservation scientists could deliver astutely framed evidence, showing how protecting biodiversity is not antithetical to other political priorities, where possible.

Despite the reservations of Pullin and Knight (2012) and Sutherland et al. (2013), conservation scientists can achieve a “more intelligent engagement with politics” (Jasanoff 2013:63); Balmford (2012) and Avery (2012) convey the benefits of doing so. Whilst scientific and technical rigor remain vital, it is unwise in a mission-driven pursuit to assert that evidence is value free. Conservation scientists are rarely “objective purveyors of knowledge” (Takacs 1996:190) because they care about influencing policy to help nature. Therefore, conservation scientists must not be afraid to innovate by identifying salient political contexts; after all, “to think up new and better methods of arguing in any field is to make a major advance” (Toulmin 1958:257).
